# Vitamin D Metabolites and Sex Steroid Indices in Postmenopausal Women with and without Low Bone Mass

**DOI:** 10.3390/metabo11020086

**Published:** 2021-02-01

**Authors:** Nasser M. Al-Daghri, Sobhy M. Yakout, Mohammed G.A. Ansari, Syed D. Hussain, Kaiser A. Wani, Shaun Sabico

**Affiliations:** Chair for Biomarkers of Chronic Diseases, College of Science, King Saud University, Riyadh 11451, Saudi Arabia; syakout@ksu.edu.sa (S.M.Y.); mansari@ksu.edu.sa (M.G.A.A.); shussain@ksu.edu.sa (S.D.H.); kwani@ksu.edu.sa (K.A.W.); ssabico@ksu.edu.sa (S.S.)

**Keywords:** vitamin D metabolites, osteoporosis, osteopenia, Saudi postmenopausal women

## Abstract

While the independent roles of vitamin D and sex hormones in skeletal health are well established, the associations of vitamin D and its metabolites to sex hormones and their indices are less investigated. In this observational study, clinical information of 189 Saudi postmenopausal women aged ≥50 years old [N = 80 with normal bone mineral density (BMD), aged 53.3 ± 7.7 years with body mass index (BMI)= 34.1kg/m^2^ ± 5.8, and N = 109 with low BMD (T-score −1.0 to −2.5), aged 57.0 ± 8.2 years, BMI = 32.4kg/m^2^ ± 6.2] was extracted from an existing capital-wide osteoporosis registry in Riyadh, Saudi Arabia. Data included were BMD scores, serum total 25(OH)D, sex hormones, and bone turnover markers which were measured using commercially available assays. Age- and BMI-adjusted comparisons revealed significantly higher parathyroid hormone (PTH) levels as well as significantly lower testosterone and bioavailable testosterone in the low BMD group than the normal BMD group (*p*-values 0.04, 0.02, and 0.03, respectively). Stepwise linear regression showed that circulating testosterone levels accounted for 9.7% and 8.9% of the variances perceived in bioavailable 25(OH)D and free 25(OH)D, respectively (*p* < 0.01), independent of other sex hormones, sex hormone indices, and bone turnover markers. Our study suggests that androgens are significantly associated with non-conventional vitamin D metabolites and these associations may have clinical relevance in assessing risk for low BMD and osteoporosis in Arab postmenopausal women.

## 1. Introduction

25-hydroxy vitamin D (25(OH)D) has an established role in calcium homeostasis for optimal skeletal health. In recent decades and in the past year alone, scientific advances continue to provide accumulating evidence of the pleiotropic effects of 25(OH)D on overall human health and its associations with extra-skeletal functions and clinical conditions [1,2,3]. Vitamin D deficiency is affecting people globally and is deemed as a risk factor for many chronic diseases [4,5], especially osteoporosis in adults [6,7] and rickets in children [8]. While the Kingdom of Saudi Arabia is blessed with ample sunlight year-round, 25(OH)D deficiency remains widespread in the population [9,10]. Furthermore, the prevalence of osteoporosis is at 34–39.5% for 50–80-year-old women and 21.4–30.7% in healthy men [11,12].

In humans, 85–90% of 25(OH)D is linked to vitamin D binding protein (VDBP), whereas the remaining 10–15% are linked to albumin and lipoproteins, and the rest of the <0.03% are considered free [13]. The sum of free and albumin-bound 25(OH)D represents bioavailable 25(OH)D which reflects the level of 25(OH)D that is readily accessible for cells or tissues [14]. In line with the “free-hormone hypothesis,” only the free-hormone fractions can go to cells and produce biological activates [15,16].

It has been established that skeletal health is largely influenced by endogenous sex hormones in both men and women, with estrogen levels largely dictating the extent of bone loss in women in both their reproductive and post-menopausal stages [17,18,19]. While testosterone, another sex hormone, also has significant, albeit indirect effects in bone health among men in particular, its low levels and associated risk in bone-related diseases such as osteoporosis are largely due to lesser substrate availability for the conversion of estradiol, which is the main bone health determinant in both sexes [18,20]. The carrier of these hormones, known as the sex hormone binding globulin (SHBG), which is mainly produced by the liver, is bound to sex hormones for transport to the target cells where they exert their biological activities [21]. Like the sex hormones, SHBG levels have also been implicated in bone diseases, but mostly age-related and limited to select skeletal sites [22].

Despite the established independent relationships of vitamin D and sex hormones in human skeletal health, there are limited studies determining the associations of vitamin D and its metabolites to sex hormones and their indices. Such studies may shed light on which among the various vitamin D metabolites exert major influence on androgen physiology which can, in turn, translate to better risk assessment and management of patients having low bone density issues and associated osteoporosis risk. The present cross-sectional study aims to fill this gap and determine such associations among postmenopausal Arab women with or without established low bone density.

## 2. Results

The general characteristics of all 189 subjects with low bone mineral density (BMD) and normal BMD are given in Table 1. Low BMD was observed in 57.7% of participants. The low BMD group were significantly older and had significantly lower BMI than those with normal BMD (*p* values <0.001). Worthy to note was that the mean BMI in both groups fell in the obese range. Age- and BMI-adjusted comparisons revealed that the normal BMD group had a significantly earlier onset of menarche (*p* = 0.006), older age at first pregnancy (*p* = 0.005), and shorter duration of amenorrhea (*p* = 0.01) than those in the low BMD group. As expected, T-scores and BMD values were significantly higher in the normal BMD group than the low BMD group (adjusted *p*-values <0.001). No significant difference was observed in the mean duration of menopause after adjustments (Table 1).

Table 2 shows the hormonal profiles of participants according to BMD status. Unadjusted comparisons showed that the low BMD group had significantly higher 25(OH)D levels than the normal group (*p* < 0.001) as well as higher levels of PTH (*p* = 0.002), SHBG (*p* = 0.007), and FSH (*p* = 0.002). The normal BMD group on the other hand had significantly higher levels of testosterone (*p* = 0.002), estradiol (*p* = 0.02), free androgen index (FAI) (*p* = 0.002), free estradiol index (FEI) (*p* = 0.007), free testosterone (FT) (*p* = 0.003), and bioavailable testosterone (BT) (*p* = 0.003) than the low BMD group. The majority of the parameters lost statistical significance after age and BMI adjustments were done with the exceptions of PTH, being significantly higher in the low BMD group as compared to the normal group (*p* = 0.04), and having significantly lower testosterone (*p* = 0.02) and BT levels as well (*p* = 0.03) (Table 2).

Table 3 shows the age-and BMI-adjusted comparisons in bone turnover markers in both groups and revealed no significant differences with the exception of N-telopeptides of type I collagen (NTX) which were observed to be modestly higher in the normal BMD group than the low BMD group at borderline significance (*p* = 0.05). The rest of the comparisons are found in Table 3.

Bivariate associations between vitamin D metabolites, sex hormones and bone turnover markers in all participants are presented in Table 4. No significant associations were seen between total 25(OH)D and all parameters of interest with the exception of FSH which had modest correlation (r = 0.17; *p* < 0.05).

Stepwise linear regression using the different vitamin D metabolites as dependent variables and sex hormones with bone turnover markers as independent variables revealed that testosterone (ng/mL) explained 9.7% and 8.9% of the variances perceived in bio-available 25(OH)D and free 25(OH)D, respectively (*p* < 0.01). No significant predictor was seen in total 25(OH)D (not included in tables).

## 3. Discussion

The present study determined the associations of 25(OH) D metabolites with sex hormone indices as well as bone turnover markers among Arab postmenopausal women and found that sex hormones, testosterone in particular, are significantly associated with free and bio-available 25(OH)D but not with total 25(OH)D, independent of other metabolic markers assessed. Other findings indicated that in both normal and low BMD participants, both bioavailable and free 25(OH)D correlated inversely with SHBG and positively with other sex hormones including testosterone, bioavailable testosterone (BT), free testosterone (FT), estradiol, free androgen index (FAI), and free estradiol index (FEI). The correlation of 25(OH)D with BMD may in part be attributed to sex hormone levels that were also related to 25(OH)D metabolites. SHBG is a glycoprotein that binds sex steroids with high affinity, regulating their bioavailability and entry to target cells [21]. SHBG play a role in numerous diseases, containing osteoporosis in postmenopausal women [22].

In our study, there is a significant inverse association between SHBG and both free and bioavailable 25(OH)D in both healthy and low BMD groups. Few studies examined this correlation in women, and only one study stated that there was a significant relationship between 25(OH)D deficiency and SHBG [23] This association, especially in the low BMD group, suggests an increased risk of osteopenia and osteoporosis for people with high SHBG who also have low free and bioavailable 25(OH)D not total 25(OH)D. The mechanisms for this interaction are unclear. Generally, a high 25(OH)D level may reinforce the correlation with SHBG, either through increased androgen synthesis [24] or through independent pathophysiological mechanisms [25].

FSH is important in women’s ovarian functions and an inverse correlation between FSH and 25(OH)D suggests a reduction in primordial follicles which may lead to early menopause. As a result, low 25(OH)D levels may have opposing influences on ovarian reserve in postmenopausal women. It was found that 25(OH)D was inversely correlated to urinary FSH levels in post-menopausal women.

Pilz et al. found that 25(OH)D supplementation (3332 IU /day) for 1-year increased total, free, and bioavailable testosterone levels [24], though another study found that 25(OH)D supplementation was not related to increased testosterone levels [26]. In our study, we found a significant positive relationship of 25(OH)D with total, bioavailable, and free testosterone. Although both low total testosterone and low 25(OH)D levels can be signs of poor health status, there is a causal role between 25(OH)D and testosterone production. 25(OH)D exerts its influence on testicular Sertoli cells causing increased testosterone production.

Estradiol was positively associated with free and bioavailable 25(OH)D in both healthy and low BMD groups. Low estradiol level is one of the most important factors that cause low VDBP levels in older women. Our results suggest that high estradiol is associated with higher VDBP levels. High serum VDBP occurs only in post-menopausal women with high estradiol levels. This might suggest that the interaction of estrogen with 25(OH)D depends on DBP and menopausal status.

Another interesting finding in the present study was the significant correlation of 25(OH)D with FAI and FEI, signifying that 25(OH)D might play a part in androgen bioavailability in postmenopausal women. Prospective investigations may further shed light on the role of 25(OH)D in the bioavailability of androgens and its relation to BMD in postmenopausal women. The correlation between 25(OH)D and sex hormone in our study confirms the appropriateness of 25(OH)D supplementation in postmenopausal women as a stimulator of endogenous 25(OH)D production. Furthermore, our results acknowledge that 25(OH)D supplementation in postmenopausal women might have an effect on bone metabolism, as well as on other body functions related to sex hormones actions.

The authors acknowledge several limitations. The cross-sectional design limits the findings to being at best, suggestive, and therefore causality between the relationships between 25(OH)D bioavailability and sex hormones needs to be further investigated prospectively. Several major confounding variables for vitamin D such as season, sunlight exposure, intake of vitamin D supplements, and lifestyle were not taken into consideration and may affect the findings. The lack of information with respect to vitamin D intake is vital and may partially explain the higher 25(OH)D levels in the low BMD group, although previous observational studies from the same ethnic group demonstrate a significant positive association between age and vitamin D status among adults.

## 4. Materials and Methods

### 4.1. Subjects

In this cross-sectional study, a total of 189 Saudi postmenopausal women aged ≥50 years old (N = 80 healthy control, N = 109 with low BMD) were randomly selected from the existing osteoporosis registry of the Chair for Biomarkers of Chronic Diseases (CBCD) in King Saud University (KSU), Saudi Arabia. In brief, this registry is a capital-wide database involving more than 1500 participants from the outpatient clinics of King Fahd Medical City, King Salman Hospital, and King Saud University Medical City, Riyadh, Saudi Arabia, whose BMD measurements were assessed and recorded between 2013 and 2016 [27,28]. A generalized questionnaire was used to collect participants’ history (age, medical history, disease status, etc.). Height, weight, and blood pressure were obtained from all patients. Body mass index (BMI) was calculated (kg/m^2^).

### 4.2. Biochemical Analysis

Subjects were requested to visit their respective health centers after an overnight fast (≥ 10 h). Fasting blood samples were collected and serum extracted. Extracted serum was transferred to CBCD Biobank and stored at −80 °C until further analysis. Serum 25(OH)D and sex hormones were measured using electrochemiluminescence immunoassay (Roche Diagnostics, Mannheim, Germany). PTH was estimated by biochemical analyzer COBAS e411 (Roche Diagnostics) using commercially available kits (intra-assay and inter-assay CV’s of 2.7% and 6.5%, respectively). SHBG was measured with Roche Elecsys modular analytics Cobas e411 using an electrochemiluminescence immunoassay where the lower detection limit of this assay was 0.35 nmol/L and the intra-assay CV was 2.6–5.6%. FSH, testosterone, and estradiol were also quantified using the Elecsys Cobas e-411 commercially available kits. The limits of detection for FSH, testosterone, and estradiol were 0.100 mIU/L, 0.025 ng/mL, and 5 pg/mL, respectively. Serum 25(OH)D was measured using Roche Elecsys modular analytics (Cobas e411) and assessed using electrochemiluminescence immunoassays (Roche Diagnostics, GmbH, Mannheim, Germany). Intra- and inter-assay CV were 4% and 6.5%, respectively. Serum VDBP was determined by ELISA (R&D Systems) with inter-assay CV (1.6–3.6%), and recovery of (98–103%). Free and bioavailable 25(OH)D were calculated using the concentrations of total 25(OH)D, DBP, and albumin as previously described [29]. Vitamin D deficiency was defined as 25(OH)D <50 nmol/L based on regional guidelines. [30].

### 4.3. Anthropometrics and DXA

BMD (g/cm^2^) was recorded for all selected participants at the lumbar spine and femur using Prodigy- (GE Healthcare, Chicago, IL, USA), dual-energy X-ray absorptiometry (DXA) scans. T-Scores were recorded and those below −1.0 to −2.5 indicated low bone mass based on national and regional guidelines [31,32].

### 4.4. Calculations Used

The following indices were calculated as follows:

FAI = Total testosterone/SHBG × 100

Free estrogen index (FEI) was calculated as:

FEI = Total estradiol/SHBG × 100

### 4.5. Statistical Analysis

SPSS version 16.5 (SPSS Inc., Chicago, IL, USA) was used for data analysis. Normal continuous variables were presented as mean ± standard deviation and non-normal variables were presented as median (1st quartile–3rd quartile). Non-normality was tested using histogram and confirmed using Kolmogorov–Smirnov test. Significance differences were tested using independent sample t-test and Mann–Whitney U test for normal and non-normal variables, respectively. Analysis of covariance (ANCOVA) was used to determine significant differences after adjusting for age and BMI. Spearman correlation coefficient was used to determine the relationship between study parameters. Furthermore, multivariate stepwise linear regression was used to identify variables independently associated with free, bioavailable, and total 25(OH)D. Significance was set at *p* < 0.05.

## 5. Conclusions

To the best of our knowledge, this is the first study to determine the relationship of 25(OH)D metabolites with sex hormones and their indices among postmenopausal Arab women. Androgens, particularly testosterone, are associated with bioavailable and free 25(OH)D, independent of other hormones, including bone turnover markers. The results of this study suggest the clinical importance of other vitamin D metabolites related to hormonal regulation and associated osteoporosis risk among Arab post-menopausal women.

## Figures and Tables

**Table 1:** General Clinical Characteristics of Participants according to Normal and Low Bone Mineral Density (BMD) Groups.

Clinical Parameters	Normal	Low BMD	*p*-Values	Adj. *p*-Values *
N	80 (42.3)	109 (57.7)		
Vitamin D Deficiency (%)	36 (45.0)	43 (39.4)		
Age (years)	53.3 ± 7.7	57.0 ± 8.2	<0.001	
BMI (kg/m^2^)	34.1 ± 5.8	32.4 ± 6.2	<0.001	
Age of menarche	13.0 ± 1.4	13.3 ± 1.6	0.001	0.006
Menopause (years)	9.6 ± 11.7	11.4 ± 10.6	0.01	0.30
Age during first pregnancy	19.7 ± 4.0	19.0 ± 3.6	0.006	0.005
Amenorrhea (years)	7.1 ± 6.0	9.7 ± 7.1	<0.001	0.01
T-score (Spine)	0.0 ± 0.8	−2.1 ± 0.9	<0.001	<0.001
T-Score (Femur)	0.6 ± 0.9	−0.8 ± 1.0	<0.001	<0.001
BMD (Spine)	1.21 ± 0.14	0.9 ± 0.1	<0.001	<0.001
BMD (Femur)	1.13 ± 0.13	0.9 ± 0.1	<0.001	<0.001

Note: Data presented as Mean ± SD. * *p*-value adjusted for age and BMI; *p* < 0.05 considered significant.

**Table 2:** Hormonal characteristics of participants according to Normal and Low BMD groups.

Biochemical Parameters	Reference Ranges	Normal	Low BMD	*p*-Values	Adj. *p*-Values *
N	--	80 (42.3)	109 (57.7)	--	--
Corrected Calcium (mmol/L)	0.7–4.0	2.3 ± 0.2	2.3 ± 0.2	0.84	0.40
25(OH) D (nmol/L)	7.5–175.0	66.4 ± 34.2	73.5 ± 36.1	<0.001	0.18
Bioavailable 25(OH)D (nmol/L) #	--	26.1 (13–51)	29.1 (14–49)	0.56	0.81
Free 25(OH)D (nmol/L) #	--	30.2 (15–54.1)	33.4 (16–64)	0.42	0.62
VDBP (mg/mL) #	0.083–50.0	42.0 (7–105)	23.2 (6–106)	0.27	0.25
PTH (pg/mL) #	1.2–5000.0	10.6 (6–20)	15.4 (8–30)	0.002	0.04
SHBG (nmol/L) #	0.8–200.0	18.2 (0.4–34)	32.3 (1–57)	0.007	0.08
FSH (mIU/mL) #	0.1–200.0	30.7 (11–51)	40.6 (21–66)	0.002	0.29
Testosterone (ng/mL) #	0.02–15.0	0.7 (0.4–1)	0.6 (0.3–0.9)	0.007	0.02
Estradiol (pg/mL) #	5.0–4300.0	108.7 (43–247)	61.8 (33–196)	0.02	0.13
FAI #	--	4.3 (1–182)	1.5 (0.6–39)	0.002	0.07
FEI #	--	307.7 (142–24168)	182.2 (62–3988)	0.007	0.09
FT (nmol/L) #	--	0.06 (0.04–0.17)	0.0 (0.0–0.1)	0.003	0.20
BT (pmol/L × 10^−4^) #	--	2.8 (2–9)	2.0 (1–4)	0.002	0.03

Note: -- indicates none; # indicates non-normal variables; VDBP, vitamin D binding protein; PTH, parathyroid hormone; SHBG, sex hormone binding globulin; FSH, follicle stimulating hormone; FAI, free androgen index; FEI, free estradiol index; FT, free testosterone; BT, bioavailable testosterone. * *p*-value adjusted for age and BMI; *p* < 0.05 considered significant.

**Table 3:** Bone Turnover Markers of Participants according to Normal and Low BMD Groups.

Biochemical Parameters	Reference Ranges	Normal	Low BMD	*p*-Values	Adj. *p*-Values *
N	--	80 (42.3)	109 (57.7)	--	--
P1NP (ng/mL) #	5–1200	11.2 (5.0–30.0)	13.4 (5.0–27.5)	0.44	0.20
CTX (pg/mL) #	10–6000	10.0 (10.0–84.5)	20.0 (10.0–75.0)	0.42	0.16
NTX (nmol/L) #	20–3000	56.2 (42.6–75.5)	49.8 (35.5–65.2)	0.06	0.05
bALP (nmol/L)	--	1.1 ± 0.3	1.1 ± 0.3	0.98	0.75

Note: -- indicates none; # indicates non-normal variables; P1NP, (Procollagen 1 Intact N-Terminal Propeptide); CTX, carboxy-terminal collagen; NTX, N-telopeptides of type I collagen; bALP, bone-specific alkaline phosphatase; * *p*-value adjusted for age and BMI; *p* < 0.05 considered significant.

**Table 4:** Correlation between vitamin D metabolites and measured parameters.

Parameters	Total 25(OH)D			Bioavailable 25(OH)D			Free 25(OH)D		
	All	Normal	Low BMD	All	Normal	Low BMD	All	Normal	Low BMD
SHBG	0.13	0.07	0.02	−0.27 **	−0.30 *	−0.26 **	−0.21 **	−0.24	−0.20 *
FSH	0.17 *	0.16	0.08	−0.12	−0.23	−0.06	−0.09	−0.09	−0.08
Testosterone	0.10	0.21	0.04	0.38 **	0.45 **	0.34 **	0.37 **	0.44 **	0.33 **
BT	0.04	0.14	0.07	0.38 **	0.48 **	0.35 **	0.34 **	0.41 **	0.32 **
FT	−0.01	0.11	0.05	0.33 **	0.44 **	0.30 **	0.34 **	0.46 **	0.31 **
Estradiol	0.01	0.03	0.06	0.39 **	0.46 **	0.34 **	0.45 **	0.46 **	0.43 **
FAI	−0.05	0.04	0.03	0.34 **	0.43 **	0.32 **	0.29 **	0.35 **	0.27 **
FEI	−0.06	−0.04	0.05	0.35 **	0.41 **	0.33 **	0.38 **	0.40 **	0.37 **
P1NP	0.07	0.13	0.03	−0.40 **	−0.35 **	−0.42 **	−0.41 **	−0.36 **	−0.43 **
CTX	0.09	−0.01	0.12	−0.23 **	−0.33 **	−0.19	−0.24 **	−0.35 **	−0.18
NTX	−0.06	−0.09	−0.03	−0.05	−0.16	−0.01	−0.07	−0.19	−0.02
bALP	−0.08	−0.16	−0.02	−0.11	−0.36	0.10	−0.12	−0.38 **	0.11

**Note**: Data presented as Spearman correlation coefficient; * and ** indicate significance at 5% and 1%, respectively.

## Data Availability

All data generated and analyzed in the study are included in the article.
